# aMLProt: an automated machine learning library for protein applications

**DOI:** 10.1093/bioinformatics/btaf543

**Published:** 2025-09-24

**Authors:** Ruite Xiang, Christian Domínguez-Dalmases, Albert Cañellas-Solé, Victor Guallar

**Affiliations:** Department of Life Sciences, Barcelona Supercomputing Center (BSC), Barcelona 08034, Spain; Facultat de Farmàcia i Ciències de l‘Alimentació, Universitat de Barcelona, Barcelona 08028, Spain; Department of Life Sciences, Barcelona Supercomputing Center (BSC), Barcelona 08034, Spain; Department of Life Sciences, Barcelona Supercomputing Center (BSC), Barcelona 08034, Spain; Facultat de Farmàcia i Ciències de l‘Alimentació, Universitat de Barcelona, Barcelona 08028, Spain; Department of Life Sciences, Barcelona Supercomputing Center (BSC), Barcelona 08034, Spain; Catalan Institution for Research and Advanced Studies (ICREA), Barcelona 08010, Spain

## Abstract

**Motivation:**

Machine learning tools have become increasingly common in biological research, driven by the emergence of pre-trained large language models. However, training effective models remains a complex task, since many choices influence their performance. AutoML (automated machine learning) approaches help address these challenges by streamlining the entire model development pipeline.

**Results:**

We developed aMLProt, an AutoML framework tailored specifically for protein applications, such as enzyme engineering and bioprospecting. It features a modular design, allowing each component to be used independently or in combination. Notably, aMLProt integrates 19 classifiers and 26 regressors, along with pre-trained protein language models. It also includes standalone applications proven useful for protein-related workflows. To enhance usability, aMLProt is integrated with Horus, a GUI-based application with a visual interface.

**Availability and implementation:**

aMLProt is available on https://github.com/etiur/aMLProt.git and https://doi.org/10.5281/zenodo.14971157; The aMLProt plugin is available via the official Horus Plugin Repository https://horus.bsc.es/repo/plugins/amlprot, and Horus itself can be freely downloaded from https://horus.bsc.es. Moreover, a demo of aMLProt can be found, without previous registration or download, at the horus.bsc.es/amlprot and horus.bsc.es/amlprot-suggest. The results and data from the pH optima regression model are available at: https://zenodo.org/records/15394097.

## Graphical Abstract

**Graphical Abstract:**
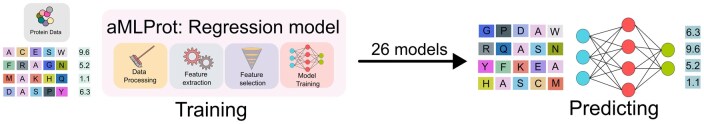

## 1 Introduction

The volume and complexity of biological datasets are increasing dramatically. When analysed effectively, these datasets can provide valuable insights to guide research. Machine learning (ML) excels at this task by identifying complex relationships in training data and applying them to make predictions about new samples.

While ML tools have become more user-friendly through open-source platforms and easy-to-use libraries, developing effective models remains challenging. Key decisions in model architecture and training can significantly impact performance, requiring expertise that many researchers lack. Even for experienced practitioners, selecting optimal algorithms and tuning hyperparameters is complex and often yields only incremental improvements (Valeri *et al.* 2023).

Additionally, randomly splitting biological data into training and test sets is problematic. Proteins and other biological samples often share phylogenetic similarity or homology, which can lead to overoptimistic test results. A model might learn to predict similar properties for homologous sequences in the test set, resulting in overfitting and poor generalization to novel data with little homology to the training set (Jones 2019).

One way to address this is by restricting predictions to data similar to the training set, a concept known as the applicability domain (Kaneko 2024). However, this would significantly limit the model’s usefulness.

To overcome these challenges and make building ML more accessible, particularly for non-experts, an automated ML (AutoML) system that streamlines the entire training pipeline with minimal user intervention would be ideal. Although several AutoML implementations exist, many are not designed to handle biological data such as protein sequences (Feurer *et al.* 2022). Some are tailored to specific tasks like function prediction (de Oliveira *et al.* 2024), or focus solely on deep learning models. For example, AutoProteinEngine (Liu *et al.* 2024b) enables users to interact with the AutoML platform via natural language, thereby lowering the barrier for users without a computational background to apply ML tools. However, simpler models, especially tree-based ones, can deliver competitive performance at a fraction of the computational cost, which is particularly relevant when working with small experimental datasets. BioAutoMATED attempts to bridge some of these gaps by integrating multiple AutoML frameworks with support for biological sequence data (Valeri *et al.* 2023), such as proteins, DNA, and RNA. It also offers a user-friendly Jupyter Notebook interface. However, it lacks support for leveraging pre-trained models as each pipeline begins training from scratch. This is a limitation, as these models, such as the ESM series, have achieved state-of-the-art performance across a range of protein-related benchmarks (Xu *et al.* 2022).

**Figure 1.**
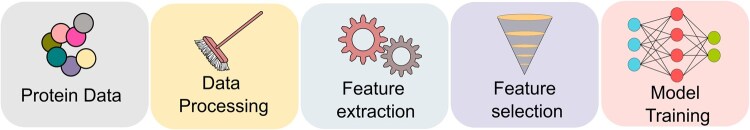
Overview of the modules implemented in aMLProt. It illustrates the AutoML pipeline, which includes modules for data preprocessing followed by feature extraction, feature selection, and model training.

With this in mind, we developed aMLProt, an end-to-end AutoML platform for protein applications such as bioprospecting and engineering. Based on our previous experience developing EP-pred (Xiang *et al.* 2022), aMLProt follows a modular structure, where each module represents key steps in the machine learning pipeline. These include data preprocessing and splitting, feature extraction and selection, outlier detection, and model selection and optimization (including pre-trained large language models) (Fig. 1). The modularity allows users to use each block independently or combine them into custom workflows, providing a high degree of flexibility. Another key focus of aMLProt was to improve the accessibility of existing machine learning tools by integrating them into built-in applications. The first tool implemented uses protein language models that have been shown to predict the functional impact of mutations (Meier *et al.* 2021). For instance, the mutations suggested by ESM models increased the binding affinity of a mature antibody by seven-fold (Hie *et al.* 2024), demonstrating its practical utility for protein optimization.

To ensure usability, aMLProt includes example Jupyter notebooks for each module and is integrated into Horus, a graphical user interface (GUI) that enables users to build and execute workflows visually, enhancing accessibility for researchers with limited programming experience.

aMLProt serves three purposes: (1) automating the often complex and time-consuming task of training ML models, (2) providing experienced practitioners with a quick way to generate baseline models for comparison with more customized and advanced protein-specific pipelines, and (3) provides immediate access to specialized tools that accelerate protein design and discovery efforts.

## 2 Materials and Methods

### 2.1 The aMLProt library organization

Here, we explain the different blocks that form the aMLProt library (Fig. 1).

### 2.2 Data preprocessing and splitting block

Protein sequences often share homology, so their splitting should be carefully tailored to the specific application. As demonstrated in *PEER: A Comprehensive and Multi-Task Benchmark for Protein Sequence Understanding* (Xu *et al.* 2022), different tasks may require different splitting strategies. A common approach is to separate training and test sets using a 30% sequence identity threshold, ensuring that sequences in the test set share no more than 30% identity with those in the training set. This minimizes the impact of sequence similarity on model performance and provides a more realistic assessment of generalizability. In some cases, such as when working with proteins from the same family, strictly enforcing this threshold may not be feasible. However, controlling sequence identity within a defined range might still be interesting to reduce its influence on model predictions.

aMLProt addresses this challenge by integrating MMseqs2 to cluster sequences based on identity. We selected MMseqs2 because it is an ultra-fast algorithm that achieves higher sensitivity at 30% identity compared to other state-of-the-art clustering methods (Hauser *et al.* 2016). Additionally, aMLProt incorporates a custom splitting class that works with these clusters, ensuring that sequences within the same cluster remain together during dataset splitting. But this class also supports custom groupings, providing additional flexibility in how sequences are divided.

### 2.3 Feature extraction block

Machines do not understand protein sequences, but they understand numbers. So, in the feature selection block, we translate sequences into numbers or vectors called features. Two packages, Possum (Wang *et al.* 2017) and iFeature (Chen *et al.* 2018), are used to extract evolutionary information and physicochemical properties, respectively. iFeature can generate 53 different types of descriptors. These features might be simplistic descriptors like the amino acid composition (AAC), which counts the frequency of each amino acid in the sequence. However, there are also more elaborate descriptors that account for the distribution, transition, or correlation of different properties, like hydrophobicity, along the sequence.

Possum generates features based on the PSSM (Position Specific Scoring Matrix) profiles that contain evolutionary information of the sequences. Although very informative, the downside of these profiles is that they depend on the length of the sequences, which hampers their direct use as features for machine learning applications. By applying different matrix transformations to make them length-independent, Possum could generate 18 different descriptors. Some transformations are inspired by sequence-based features, such as ACC, which reduces the PSSM profile from a matrix of *L* × 20 dimensions, *L* being the length of a sequence, to a vector of 20 dimensions by averaging the scores of the rows in the PSSM.

Additionally, aMLProt’s integration with Hugging Face (Wolf *et al.* 2020) allows users to leverage state-of-the-art protein language model embeddings, which have become foundational for many machine learning applications in protein research. Models like ProtTrans and the ESM family have proven effective in diverse tasks, such as thermostability predictions (Pudžiuvelytė *et al.* 2024), prediction of signal peptides (Teufel *et al.* 2022), and prediction of enzyme kinetic parameters such as Kcat, Km, and Kcat/km (Wang *et al.* 2025).

### 2.4 The PSSM generation

To generate evolutionary-based features, POSSUM was adapted to process PSSM files from PSI-BLAST (Schäffer *et al.* 2001). These files contain position-specific log-odds scores for amino acid substitutions, derived from alignments with sequences identified by PSI-BLAST. Positive scores indicate that a substitution is more frequent than random and commonly observed in the alignment, while negative scores suggest it is less favorable and evolutionarily disfavored.

Although PSI-BLAST is the standard approach to generate PSSMs, its slow processing time makes it impractical for large sequence databases. For instance, analysing the 240 000 sequences in the Lipase Engineering Database (Fischer and Pleiss 2003) took around a month. To overcome this limitation, we adapted POSSUM to work with PSSM-like files generated by MMseqs2 (Steinegger and Söding 2017), which offers a substantial speed advantage. MMseqs2 operates up to 10 000 times faster than BLAST. More importantly, it performs profile searches with the same sensitivity as PSI-BLAST but at over 400 times the speed. Like PSI-BLAST, MMseqs2 iteratively generates profiles: the first iteration performs a sequence-to-sequence search, and subsequent iterations construct a PSSM-like profile for profile-based searches. Although the methods to generate the profiles differ, both represent the likelihood of observing a specific amino acid at a given position.

By integrating MMseqs2, we reduced the processing time for 240 000 sequences from a month to under 8 h. This substantial speed improvement led to its adoption in aMLProt.

### 2.5 Feature selection block

The previous step can produce large feature vectors, many of which may be redundant or irrelevant, potentially degrading model performance and increasing training time. To address this, the feature selection block eliminates unnecessary features, ensuring a more efficient and effective model.

Feature selection methods fall into four main categories (Guyon and Elisseeff 2006):

1. Filter methods evaluate the degree of dependence between features and labels using statistical metrics like Pearson’s correlation coefficient, mutual information, *F*-score, Chi-squared, Fechner correlation, and Kendall correlation (Hopf and Reifenrath 2021).
2. Wrapper methods rely on machine learning algorithms to rank features based on their contribution to model performance, often using iterative approaches to find optimal subsets. aMLProt implements Recursive Feature Elimination (RFE) wrapped around a linear model for the selection.
3. Embedded methods integrate feature selection into the training process, typical of tree algorithms like Random Forest and XGBoost (Chen and Guestrin 2016), to determine feature importance.
4. Unsupervised methods, like principal component analysis (PCA) (Greenacre *et al.* 2022), identify features that capture the most variance or structure in the dataset without relying on labels.

aMLProt implements these methods across classification and regression tasks to optimize model performance and reduce complexity.

### 2.6 Outlier detection block

After identifying the most important features, the next step could be to remove outliers, which are data points that deviate significantly from the rest, as they can negatively impact the model’s performance. For this purpose, PyOD (Chen *et al.* 2024), the leading Python library for outlier detection, provides over 50 algorithms. These range from classic methods like LOF (Local Outlier Factor) to cutting-edge techniques such as ECOD (Empirical Cumulative Distribution Functions) and DIF (Deep Isolation Forest).

To enhance outlier detection, aMLProt combines predictions from eight PyOD algorithms: Isolation Forest, LOF, ECOD, OCSVM (One-Class Support Vector Machines), PCA, KNN (K-Nearest Neighbors), Angle-Based Outlier Detection, and Histogram-Based Outlier Detection. By identifying data points flagged as outliers by the majority of these models, aMLProt ensures robust and reliable outlier detection.

### 2.7 Model training

Finally, you can train models using the selected features from each of the selection methods, with or without the outliers. aMLProt supports comparison across 19 classifiers and 26 regressors from PyCaret (Ali 2020), using standard regression and classification metrics to help identify the most suitable pipeline and algorithms for your data.

Additionally, aMLProt integrates with the Hugging Face library, making it easy to fine-tune any available protein large language model using parameter-efficient techniques such as Low-Rank Adaptation (LoRA) (Hu *et al.* 2021) and Weight-Decomposed Low-Rank Adaptation (DoRA) (Liu *et al.* 2024a). Compared to full fine-tuning, these methods can reduce the number of trainable parameters by up to 10 000 times and cut GPU memory requirements by a factor of three. Instead of updating entire weight matrices, they keep the original weights frozen and fine-tune smaller matrices, which are later incorporated into the model, allowing for effective adaptation with minimal computational cost.

### 2.8 How to use aMLProt

To use aMLProt, users must provide two input files: a FASTA file containing the protein sequences and a label file. The label file should contain either continuous values (for regression tasks) or binary labels (0 and 1) for classification problems.

The workflow begins with feature extraction, where the FASTA file is used to generate either embeddings or other feature representations. These features are then passed to the feature selection module, if desired. Users can specify either a fixed number of dimensions or a range of dimensions for selection. The output is an Excel file in which each sheet corresponds to a different feature selection method and dimensionality setting. This Excel file can then be used as input for the outlier detection module. This module analyzes each feature set and returns a CSV file listing potential outliers, ranked by how frequently each sample was predicted as an outlier.

Since multiple feature sets are generated, aMLProt provides tools to identify the best one. Users can choose whether to exclude outliers before training. aMLProt iterates through all sheets in the Excel file, quickly training and evaluating several models using the provided labels. The output is a summary Excel file listing the top three models per feature set, ranked according to the user-selected performance metric. Sheets are ordered by the aggregate performance across all models, making identifying the best-performing feature set easy.

Once the optimal feature set is selected, users can perform more detailed comparisons of model performance and tune the top-performing algorithms by optimizing their hyperparameters. Finally, the best model can be saved and used to predict outcomes for new sequences.

One important point to note is that dataset size can strongly influence model performance, yet biological data are often limited, increasing the risk of overfitting. To mitigate this, aMLProt implements best practices recommended by Vabalas *et al.* (2019), including the use of a held-out test set, feature selection restricted to the training data, a maximum feature-to-sample ratio of 1:2, and fewer cross-validation folds, all set as default parameters.

### 2.9 Standalone applications: mutant suggestion block

aMLProt includes a mutation suggestion application that leverages protein language models compatible with Hugging Face, by default the ESM2-650M model, following approaches similar to those described in Meier *et al.* (2021).

The workflow is simple: users start by providing a FASTA file containing the wild-type protein sequence, and the module generates a CSV file listing the relative log-likelihoods for every possible amino acid substitution at each position. Positive values indicate that the language model favors the mutated residue over the wild type in the given sequence context. These high-scoring mutations are not only more likely to be tolerated but also to improve function. Previous studies have shown that such mutations can lead to increased binding affinity, drug resistance, enzyme activity, and viral fitness (Hie *et al.* 2024). By capturing patterns from millions of natural proteins, the model provides an efficient way to focus experimental efforts on mutations that are more likely to have a beneficial effect.

## 3 Results

### 3.1 The aMLProt implementation in Horus

To enhance user-friendliness, we integrated the various ML blocks into Horus (https://horus.bsc.es/). This modular workflow manager enables intuitive, drag-and-drop control of each block, similar to Galaxy (The Galaxy Community 2024). Horus supports the creation of reproducible pipelines by allowing users to define the sequence and logic of tasks through an interactive 2D canvas.

It automatically manages input dependencies and execution order, ensuring workflow integrity and efficiency. Additionally, a user-friendly Python API facilitates the development of custom blocks, making Horus adaptable to specific research needs.

In the context of aMLProt, we have developed a set of specialized blocks for each ML module. These can be combined to form a complete pipeline or used alongside other available Horus blocks to build custom workflows. To illustrate its application, we implemented two example workflows: bioprospecting and enzyme engineering.

### 3.2 Bioprospecting workflow (machine learning)

We present an example workflow within Horus for training a machine learning model to support bioprospecting. In many industrial settings, pH is a key factor, as substrate solubilization often depends on maintaining conditions within a specific pH range. Two existing models, OphPred (Zaretckii *et al.* 2024) and EpHod (Gado et al. 2024), used embeddings from pre-trained protein language models to predict optimal pH values of enzymes. Both deliver comparable performance when tested on the same held-out test set from EpHod: OphPred achieves a mean absolute error (MAE) of 0.6, while EpHod reports an MAE of 0.7.

We also trained a model in aMLProt using ESM2-650M embeddings without any feature selection, using the same training and held-out test dataset, which includes a total of 9855 enzymes with experimentally measured pH optima. The entire process was automated, including data standardization using scikit-learn’s StandardScaler and model training using five-fold cross-validation via scikit-learn’s GroupKFold, grouping sequences based on identity to ensure that the training and validation sets shared no more than 20% sequence identity.

**Figure 2.**
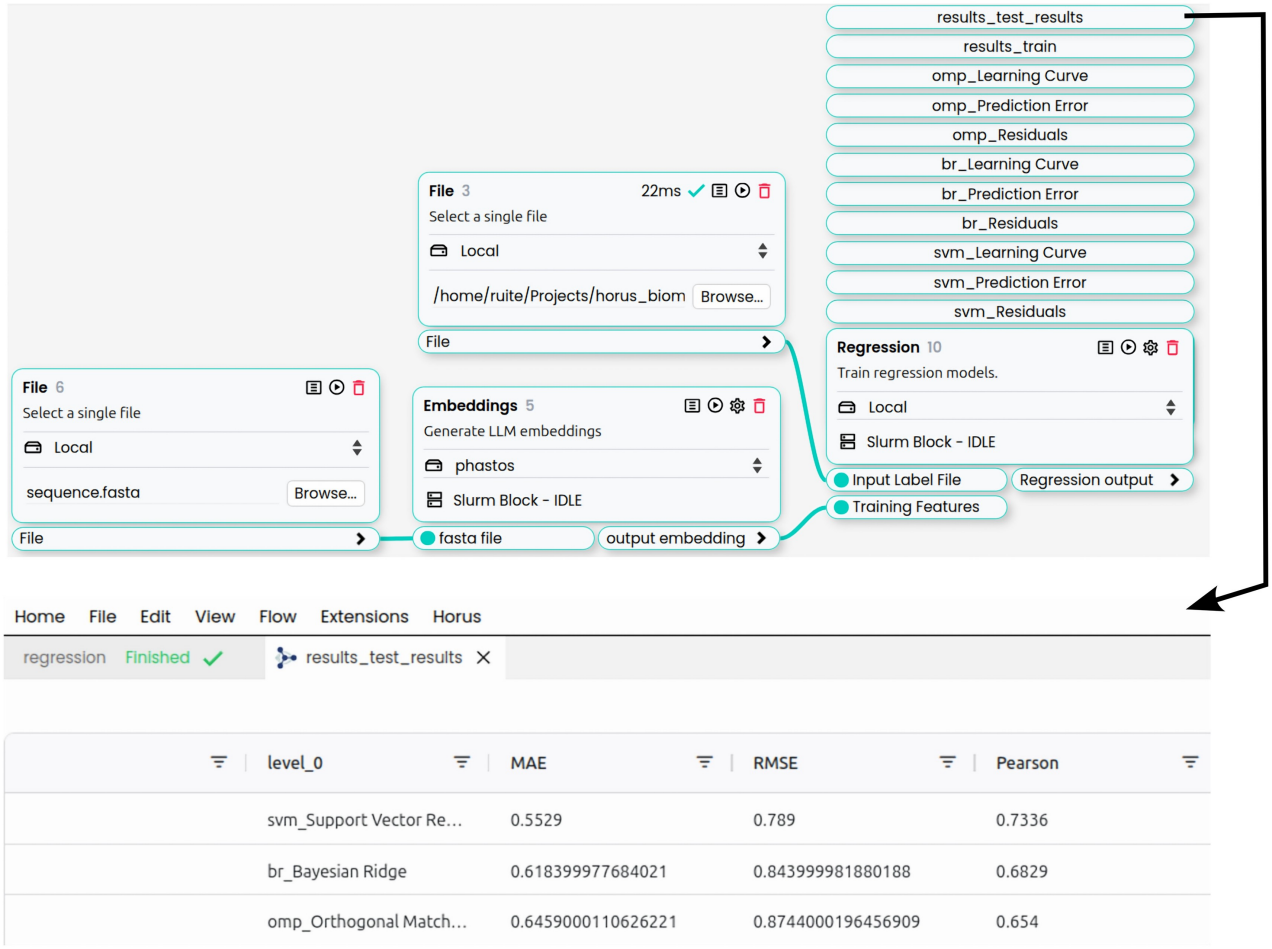
The Horus canvas with a simplified aMLProt workflow for training the pH optima predictor. Each block in the canvas represents a distinct module from the aMLProt library. The workflow starts with basic inputs, such as the sequence fasta file, which supplies the basic data. The FASTA file is passed to the Embeddings block to generate ESM embeddings. These embeddings and the corresponding labels provided as a file from a different block are then fed into the Regression block. All of the Regression block outputs are available on top of the block. The output contains the training and testing results of the top three models: support vector machine (SVM), Bayesian ridge (BR), and orthogonal matching pursuit (OMP), evaluated using metrics such as mean absolute error (MAE), root mean squared error (RMSE), and Pearson correlation. In addition, the outputs include plots such as learning curves for each model, providing a quick overview of performance trends and training behavior.

The top three models, based on the mean training and validation performance during cross-validation (Table S1, available as supplementary data at *Bioinformatics* online), were automatically selected for evaluation on the held-out test set (Table S2, available as supplementary data at *Bioinformatics* online). Within a few hours, and without any additional hyperparameter tuning (using PyCaret’s default settings), the best-performing model, SVM, achieved a MAE of 0.55, matching the performance of existing models (Fig. 2).

In addition to aMLProt, Horus supports other plugins that can seamlessly interact with aMLProt workflows. For instance, you can input a FASTA file containing the enzyme sequences for which you want to predict the pH optima. After selecting the top candidates, you can generate their protein structures using ESMFold (Lin *et al.* 2023) (https://horus.bsc.es/repo/plugins/esmfold) and then proceed to assess their activity by docking relevant substrates using RDock (Ruiz-Carmona *et al.* 2014) (https://horus.bsc.es/repo/plugins/rdock). This streamlined pipeline enables a comprehensive analysis, from sequence to structure and function, within a single platform.

### 3.3 Suggest Mutations workflow (enzyme engineering)

**Figure 3.**
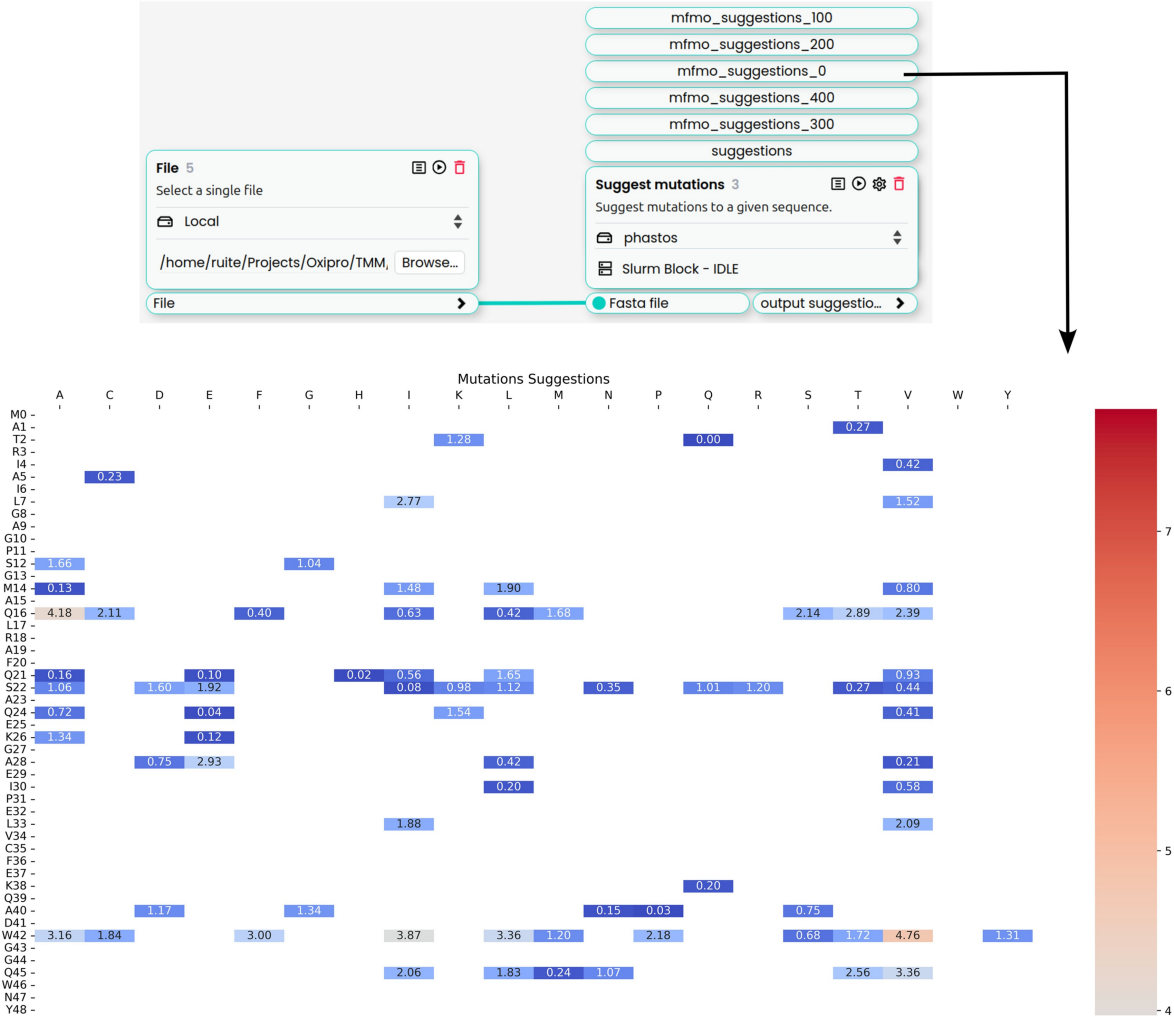
The Horus canvas with the Suggest Mutations application. The input FASTA file is provided through the File block and connected to the Suggest Mutations block. The resulting outputs are shown as clickable mini text boxes on top of the Suggest Mutations block, which open in separate tabs within Horus for easier visualization. The “suggestions” tab displays the CSV file containing the ESM relative log-likelihoods, while “mfmo_suggestions” presents the corresponding heatmaps generated from this data. The numeric values indicate the starting row within the CSV for each visualization.

The *Suggest Mutations* block is a block for enzyme engineering that requires only a FASTA file as input. It processes the sequence and outputs a CSV file containing the relative log-likelihoods computed by the ESM2-650M model for each possible amino acid substitution at every position. These values are calculated as the log-ratio of the mutant likelihood to the wild-type likelihood: log(mutant/wild-type). It also generates a series of heatmaps from the CSV file, coloring only the columns for each row with a log likelihood greater than 0.5, indicating residues that are more likely than the wild type according to the model (Fig. 3).

## 4 Conclusions

We introduced aMLProt, a user-friendly AutoML Python library designed for protein-related applications such as bioprospecting and enzyme engineering. Its integration with Horus offers an intuitive GUI, enabling users, regardless of their machine learning expertise, to interact with individual modules independently or as part of larger workflows. This modularity allows for flexible and customizable machine learning pipelines. Furthermore, the connection with other Horus plugins enables the development of more complex, multi-step applications within the same platform.

## Supplementary Material

btaf543_Supplementary_Data
