# Supplementary material for: aMLProt: an automated machine learning library for protein applications
Source: Bioinformatics. 2025 Sep 24;41(10):btaf543. doi: 10.1093/bioinformatics/btaf543 (PMC12534902; doi:10.1093/bioinformatics/btaf543)
Supplement: btaf543_Supplementary_Data [file btaf543_supplementary_data.docx]

**Supplementary Table 1.** Five-fold cross-validation results for the top seven models on the training set. The table reports the mean and standard deviation of both training and validation performance.

|  | **Split** | **Fold** | **MAE** | **MSE** | **RMSE** | **R2** | **NDCG** | **Pearson** |
| --- | --- | --- | --- | --- | --- | --- | --- | --- |
| **br** | **CV-Train** | **Mean** | 0,57 | 0,62 | 0,79 | 0,53 | 0,72 | 0,73 |
|  |  | **Std** | 0,01 | 0,01 | 0,01 | 0,02 | 0,03 | 0,01 |
|  | **CV-Val** | **Mean** | 0,68 | 0,85 | 0,92 | 0,35 | 0,62 | 0,59 |
|  |  | **Std** | 0,03 | 0,09 | 0,05 | 0,06 | 0,07 | 0,05 |
| **svm** | **CV-Train** | **Mean** | 0,35 | 0,38 | 0,61 | 0,71 | 0,80 | 0,86 |
|  |  | **Std** | 0,01 | 0,01 | 0,01 | 0,01 | 0,01 | 0,00 |
|  | **CV-Val** | **Mean** | 0,64 | 0,83 | 0,91 | 0,37 | 0,70 | 0,61 |
|  |  | **Std** | 0,04 | 0,12 | 0,07 | 0,05 | 0,04 | 0,04 |
| **omp** | **CV-Train** | **Mean** | 0,62 | 0,70 | 0,84 | 0,47 | 0,66 | 0,68 |
|  |  | **Std** | 0,01 | 0,01 | 0,01 | 0,01 | 0,03 | 0,01 |
|  | **CV-Val** | **Mean** | 0,71 | 0,92 | 0,96 | 0,29 | 0,62 | 0,56 |
|  |  | **Std** | 0,03 | 0,09 | 0,05 | 0,06 | 0,04 | 0,05 |
| **ard** | **CV-Train** | **Mean** | 0,57 | 0,59 | 0,77 | 0,55 | 0,72 | 0,74 |
|  |  | **Std** | 0,01 | 0,01 | 0,01 | 0,01 | 0,02 | 0,01 |
|  | **CV-Val** | **Mean** | 0,71 | 0,91 | 0,95 | 0,30 | 0,58 | 0,56 |
|  |  | **Std** | 0,03 | 0,09 | 0,05 | 0,07 | 0,06 | 0,05 |
| **gbr** | **CV-Train** | **Mean** | 0,54 | 0,54 | 0,74 | 0,58 | 0,92 | 0,78 |
|  |  | **Std** | 0,01 | 0,01 | 0,01 | 0,01 | 0,03 | 0,00 |
|  | **CV-Val** | **Mean** | 0,68 | 0,90 | 0,95 | 0,31 | 0,64 | 0,56 |
|  |  | **Std** | 0,04 | 0,12 | 0,06 | 0,04 | 0,05 | 0,04 |
| **ada** | **CV-Train** | **Mean** | 0,69 | 0,80 | 0,89 | 0,39 | 0,98 | 0,64 |
|  |  | **Std** | 0,01 | 0,02 | 0,01 | 0,02 | 0,02 | 0,01 |
|  | **CV-Val** | **Mean** | 0,73 | 1,00 | 1,00 | 0,24 | 0,57 | 0,49 |
|  |  | **Std** | 0,04 | 0,13 | 0,06 | 0,04 | 0,09 | 0,04 |
| **knn** | **CV-Train** | **Mean** | 0,49 | 0,49 | 0,70 | 0,63 | 0,81 | 0,79 |
|  |  | **Std** | 0,00 | 0,01 | 0,01 | 0,01 | 0,04 | 0,01 |
|  | **CV-Val** | **Mean** | 0,71 | 0,95 | 0,98 | 0,27 | 0,68 | 0,55 |
|  |  | **Std** | 0,04 | 0,10 | 0,05 | 0,05 | 0,04 | 0,04 |

**Supplementary Table 2. Performance of the top three models on the held-out EpHod test set.** *SVM* stands for Support Vector Regression, *BR* for Bayesian Ridge, and *OMP* for Orthogonal Matching Pursuit. Metrics reported include: MAE (Mean Absolute Error), MSE (Mean Squared Error), RMSE (Root Mean Squared Error), NDCG (Normalized Discounted Cumulative Gain), and Pearson (Pearson correlation coefficient).

|  | **MAE** | **MSE** | **RMSE** | **R2** | **NDCG** | **Pearson** |
| --- | --- | --- | --- | --- | --- | --- |
| **svm** | 0,55 | 0,62 | 0,79 | 0,53 | 0,6807 | 0,7336 |
| **br** | 0,61 | 0,71 | 0,84 | 0,46 | 0,6386 | 0,6829 |
| **omp** | 0,64 | 0,76 | 0,87 | 0,42 | 0,6516 | 0,654 |
